# Self-esteem levels in school-going adolescents across the slums of Karachi, Pakistan: a cross-sectional analysis

**DOI:** 10.3389/frcha.2023.1175826

**Published:** 2023-05-19

**Authors:** Hira Naeem, Sana Sharif, Hina Sharif, Tooba Seemi

**Affiliations:** ^1^ Research and Publication Department, SINA Health Education & Welfare Trust, University of Karachi, Karachi, Pakistan; ^2^ Research & Publication Department, SINA Health Education & Welfare Trust, MPH University of Saskatchewan, Karachi, Pakistan; ^3^ Research & Publication Department, SINA Health Education & Welfare Trust, Aga Khan University, Karachi, Pakistan

**Keywords:** self-esteem, adolescents, students, slum areas, parenting style, parent's literacy level

## Abstract

**Introduction:**

For individuals to live their lives and integrate into society, self-esteem is an essential feeling. Self-esteem development depends on the environment in which children are nurtured. Assessment techniques using questionnaires include Rosenberg's self-esteem scale.

**Objective:**

The study aimed to assess the self-esteem of school-going adolescents in slum areas.

**Methodology:**

This school-based cross-sectional study was conducted in three understudied slum areas of Karachi, Pakistan. A standardized scale, the Rosenberg Self-esteem Scale (RSES), and a pre-tested demographic scale was used to assess the impact of gender, weight, academic performance, tuition, and parent's education level along with parent's strictness on the self-esteem of the understudied population of adolescents aged between 11 and 19 years included in the study. Parent consent was obtained before visiting the schools.

**Findings:**

As per the collected data on self-esteem, among 539 school-going adolescents, 232 (43%) were male and 307 (57%) were female. Most students, 324 (60%), were in the 14–16 age range. Parents' education status and strictness towards their children, academic performance, and adolescent body mass index (BMI) influenced self-esteem levels.

**Conclusion:**

The study found that age, parent's education, parent's strictness, BMI, and academic performance were linked to the levels of self-esteem in the target population regardless of gender. Children's surroundings play an imperative role in developing lower or higher self-esteem in children. Assessing adolescent's self-esteem can be a useful way to build strong self-confident youngster and also beneficial to treatment for those with psychosomatic complaints in their growing age.

## Introduction

Self-Esteem greatly impacts adolescents' academic performance (1). Numerous definitions of self-esteem have been developed over time. According to Rosenberg, one's attitude about themselves, whether positive or negative, directly affects how satisfied one feels with their life (2). Positive self-esteem shields children and adolescents from mental discomfort and despondency and enables them to cope appropriately with challenging and stressful life events (3). When entering school, a child develops a broad assessment of self-worth. The degree to which adolescents experience success and failure in their lives influences how they feel about themselves. This value is based on the number of activities in which a child excels or fails, as well as the feedback from parents regarding performance (4).

Different studies have been conducted related to self-esteem assessment among children and adolescents. In the USA, researchers assessed the relationship between self-esteem and academic achievement and found that these are significantly linked (5). Another study in Bangladesh found that academic achievement is correlated with positive self-esteem and self-study (19). Gender differences also show a different kind of pattern in self-esteem. One study conducted in the USA and England (6) found that girls have lower self-esteem than boys at their adolescent age.

Similarly, another study conducted in Canada also found the same results: boys have higher self-esteem than girls (7). However, researchers from India found no influence of gender on self-esteem (8). Moreover, different meta-analyses found an association between parenting style and self-esteem. According to a popular notion, authoritative parenting increases children's self-esteem, whereas authoritarian and controlling parenting have the opposite effect (9–11). Parents may encourage social skills by modeling kind and encouraging behavior, which promotes positive relationships with others and serves as a source of self-esteem (12). Research from Bangladesh also found that parenting is imperative in growing children's and adolescents' self-esteem (13). Systematic reviews and meta-analyses also demonstrate the impact of obesity on many aspects of psychological health, such as self-esteem and body image (14). Compared with healthy-weight peers, self-esteem is lower in adolescents with obesity (15). Past research by Dubow et al. in 2009 demonstrated that parental education is a powerful positive predictor of children's development (16). Another factor that can contribute to the development of self-esteem is attending private tutoring. According to a study in Nepal by Khim Raj Supedi (2018), private tutoring has positive consequences in developing self-confidence through improved learning and immediate support from teachers (17).

Minimal studies have been conducted in Pakistan to examine self-esteem among adolescents (18). One study found that adolescents from urban areas showed comparatively more self-esteem than the rural population. In addition, male adolescents showed higher self-esteem than female adolescents.

## Study rationale

Education offers an opportunity for society and its people to be empowered. It is a tool for socioeconomic development. Education enhances social, sentimental, and psychological advancement and society. However, only some have profited from the advantages of the current education system. In this context, slum children's education in the country remains a major topic of concern. A diverse range of factors contributes to the absence of more than half of Pakistan's school-age children. Pakistan faces a significant challenge in ensuring that all children attend, stay, and learn in schools, especially the most vulnerable. While enrollment and retention rates are improving, progress in enhancing learning indicators in Pakistan could be more active. A predicted 22.8 million children and adolescents aged 5–16 are not attending school (20, 21).

## Objectives

This study aims surround the following six objectives:

1. To determine the gender-based analysis regarding the levels of self-esteem among school-going adolescents of three highly densely inhabited slum areas of Karachi, Pakistan.
2. To assess the impact of BMI on the self-esteem of adolescents of Karachi, Pakistan.
3. To assess the impact of academic performance on the self-esteem of adolescents of Karachi, Pakistan.
4. To assess the impact of parents' education on the self-esteem of adolescents of Karachi, Pakistan.
5. To assess the impact of parents' strictness on the self-esteem of adolescents of Karachi, Pakistan.
6. To assess the impact of tuition on the self-esteem of adolescents in slum areas of Karachi, Pakistan.

## Hypothesis

On the basis of the prior research discussed in the literature, the following research hypotheses were formulated for the present study:

H1: There would be a significant impact of gender on the self-esteem of school-going adolescents.
H2: There would be a significant effect of the BMI of adolescents from slum areas of Karachi, Pakistan, on their self-esteem.
H3: There would be a substantial impact of parent's education on the self-esteem of adolescents from slum areas of Karachi, Pakistan.
H4: There is a significant contribution of parent's strictness to the self-esteem of adolescents from slum areas of Karachi, Pakistan.
H5: There is a noteworthy impact on the self-esteem of adolescents from slum areas of Karachi who are attending tuition centers along with their regular school.
H6: There is a noteworthy association between the self-esteem and academic performance of adolescents from slum areas of Karachi, Pakistan.

## Methodology

### Study site

**Figure 1:**
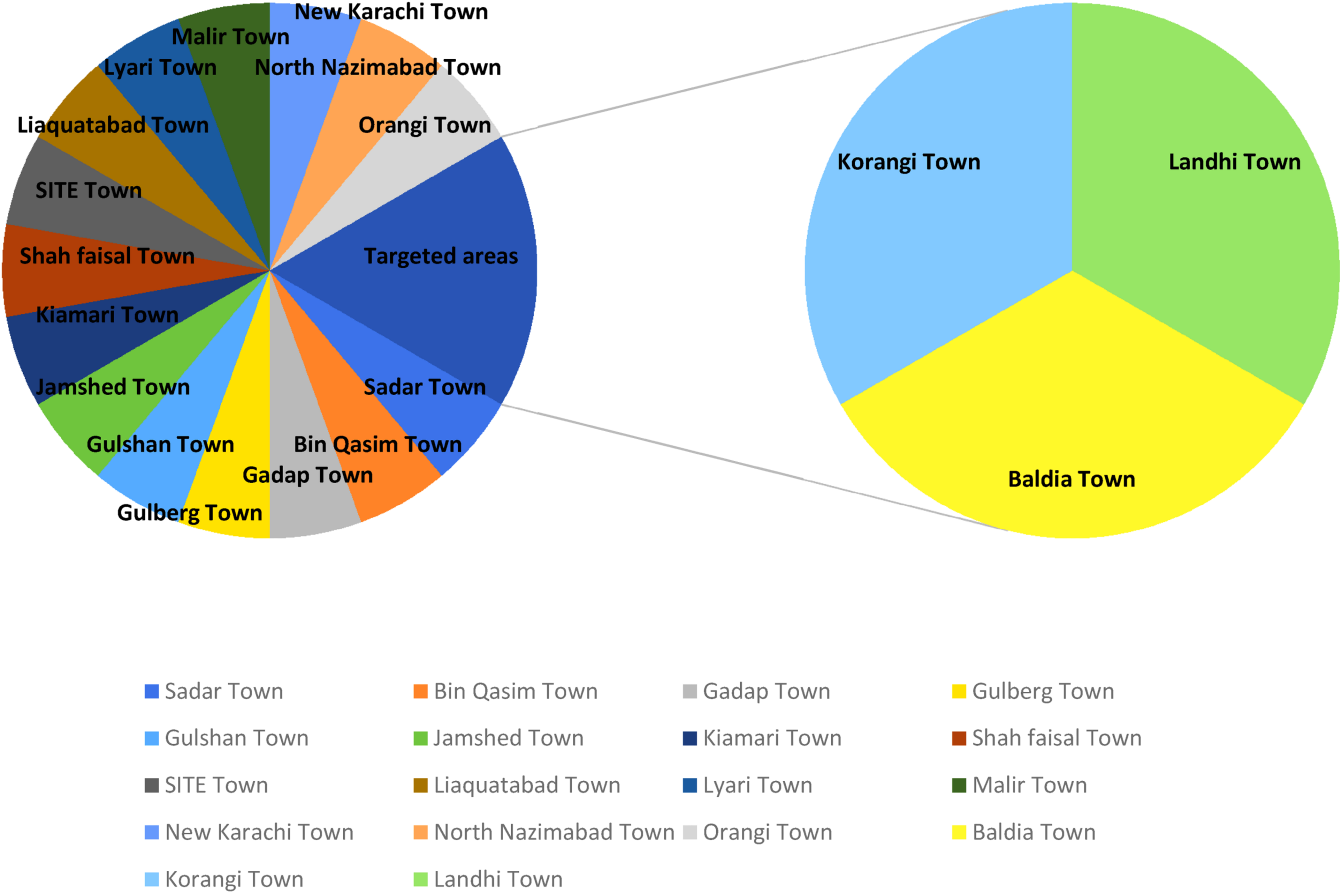
Different towns of Karachi city.

This school-based cross-sectional study was conducted in three large slum areas of Karachi: Landhi district, Korangi district, and Baldia Town. Eighteen towns in Karachi contain nearly 70% of the slums (22). Some of the major towns are shown in Figure 1, along with the targeted towns where the schools for this study were accessed to assess self-esteem among children and adolescents.

### Data collection tools

Two scales were used to collect the required data from children and adolescents.

### Demographics

The initial stage was to collect demographic information from participants, such as age, gender, education, income of their parents, and residential location. Parent's strictness was assessed by asking if any of the parents were strict and, if the respondent answered “yes”, a follow-up question asking which parent was stricter. Parent's education was measured by asking for the father's or mother's highest level of educational attainment.

### Rosenberg self-esteem scale (RSES)

In second part of the study, participants completed the Rosenberg self-esteem scale to assess self-esteem. The RSES consists of a 10-item questionnaire self-report that assesses overall perceptions of worth and acceptance. The respondent was required to directly state their feelings about themselves on all ten items. The evaluation is done on a four-point scale: strongly agree, agree, disagree, and strongly disagree—1, 2, 3, and 4, respectively—for positive items, and scored in reverse for negative items (23). The range for the total score is 10–40. A higher scores shows a higher level of self-esteem. Numerous studies have found that the RSES has strong validity and reliability (24). It has a unidimensional factor structure (25). The RSES scale was translated from English to Urdu before being administered to the targeted population to prevent language barrier issues and allow participants to understand the questions to fulfil the study objectives. The research associates and data collectors collected the data face-to-face.

### Participants of the study

Adolescents aged 11–19 years from three slum locations in Karachi, Pakistan, were the targeted participants of the study.

### Inclusion and exclusion criteria

**Figure 2:**
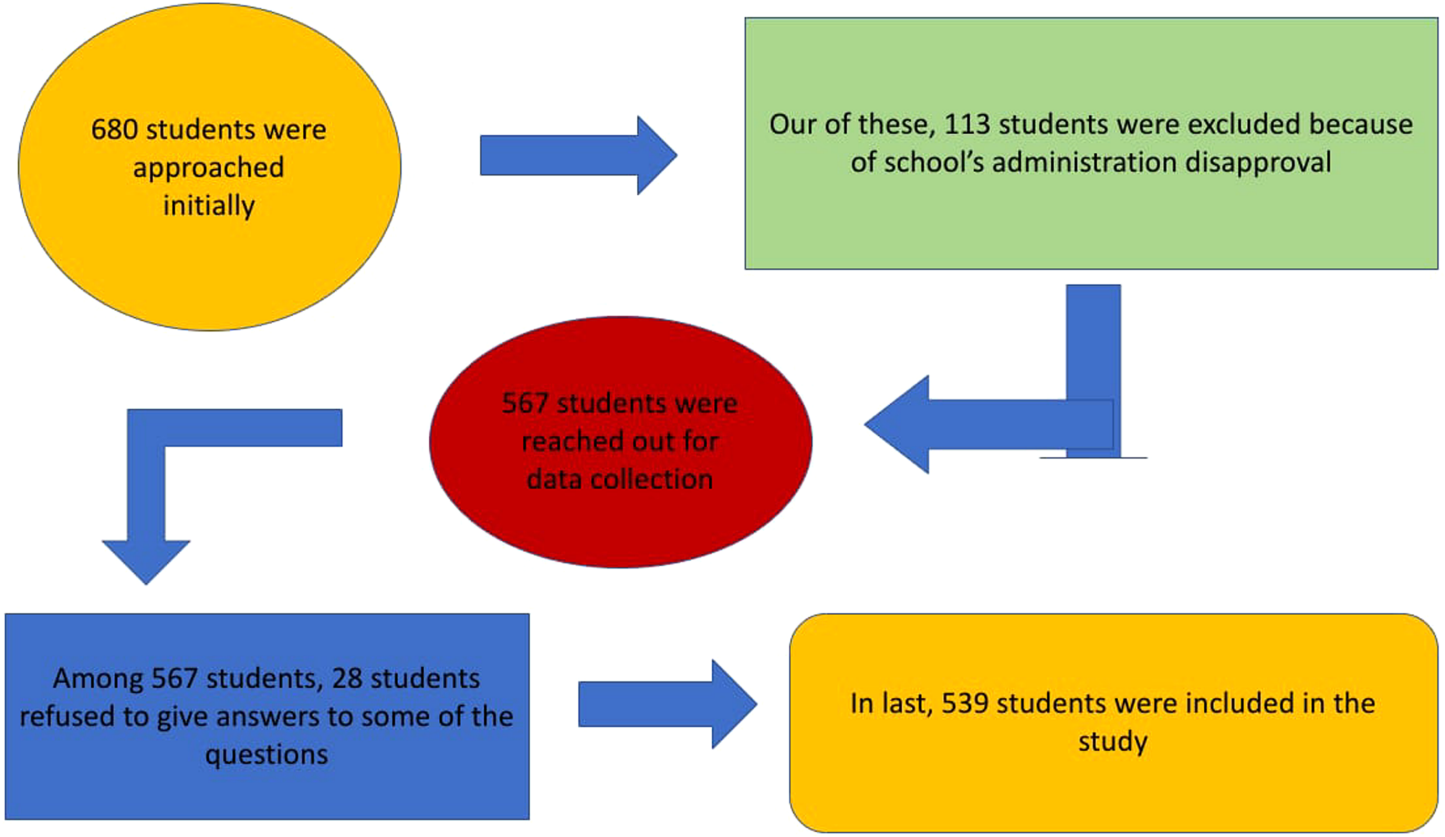
Inclusion and exclusion criteria for participating children and adolescents of slum areas of Karachi, Pakistan.

Adolescents were included in the study after their parent's written consent to participate in the Study. Those who were suffering from any identified psychological or suffered from disease or were reluctant to answer any question were excluded from the Study. Diagrammatic illustration of inclusion and exclusion of study subjects depicted in Figure 2.

### Data size, study duration, and data size calculation

Out of the total schools in the targeted towns, three schools (government and private) were included in the study using a random number table. A sample of five hundred thirty-nine (539) children and adolescents of 11-19 years of age was selected after approval was obtained from the SINA-Ethical Review Board SINA-ERB. The data was collected in two months' time period i.e October - November 2022. Since the population at large was unaware of how self-esteem is determined, especially in slum areas, we assumed the prevalence as 50% as there is no such literature related to self-esteem prevalence in Pakistan, with 5% absolute precision, a 95% degree of confidence. and adding 20% of non-response rate with ±7 patients of missing response on the questionnaire. The final data size inflated to 539.

Patientsamplesize=(4pq/d2)×20%NRR(noresponserate)

where *p* = anticipated the prevalence, *q* = (1 − *p*), *d* = margin of error, *d* = 5%

Patientsamplesize=((4∗50∗(1−50))/(5∗5))∗20%

Patientsamplesize=392∗20%=470+23=493±7=500

### Statistical analysis

Before being entered into Microsoft Excel 2007 and exported to SPSS version 24 for analysis, all collected data were checked for accuracy and uniformity. To characterize the self-esteem, clinical variables, and sociodemographic characteristics, the data were summarized through descriptive statistics (frequencies, tables, percentages, and averages). To analyze the mean differences in the self-esteem levels of students, the independent sample t-test was used on basis of gender (male vs. female), age, parents strictness (father vs. mother), having tuition (yes vs. no), BMI (normal weight vs. overweight), parents' education level (educated vs. uneducated), and the academic performances of students (below the average vs. above the average). In order to establish whether there was statistical support that the associated population means are statistically significantly different, we used this test to obtain an exact representation of the sample by comparing the means of two independent groups.

## Results

We conducted independent sample t-tests to estimate the mean differences among the different study groups. The descriptive analysis of study variables summarized in Table 1 represents the total number of participants and the percentages. The total number of female participants, 307 (57%), was higher than male participants, 57 (43%). There was a very limited number of high levels of self-esteem present in our studied population. Adolescents aged 11–19 years participated in the study. The 14–16 age range contributed to more than half of the total population (324; 60%). After calculating BMI from the height and weight of respondents, we found this significantly affected the levels of self-esteem. There were 341 (63%) overweight children and 198 (37%) of normal weight. Parents of most of the children were not educated, 338 (63%), whereas only 201 (37%) parents had some education. In addition, 210 (39%) students were above the average in academic performance while 329 (61%) were below the average. In total, 212 (39%) students were those who take tuition after their school. Additionally, 344 (64%) children and adolescent had strict fathers and 195 (36%) had strict mothers.

**Table 1:** Descriptive statistical analysis of demographic characteristics of the study sample (*N* = 539).

Variables	Total	%
**Gender**		
Male	232	43
Female	307	57
**Age**		
11 years–13 years	153	28
14 years–16 years	324	60
17 years–19 years	62	12
**BMI**		
Normal weight	198	37
Overweight	341	63
**Parent's education level**		
Educated	201	37
Uneducated	338	63
**Ethnicity**		
1. Urdu speaking	380	71
2. Punjabi	65	12
3. Sindhi	9	2
4. Balochi	7	1
5. Pathan	50	9
6. Others	28	5
**Who is strict?**		
Father	344	64
Mother	195	36
**Do you take tuition?**		
Yes	212	39
No	327	61
**Academic performance**		
Below the average	329	61
Above the average	210	39

In our collected data, we found out that there was a significant association between the level of self-esteem and weight, the strictness of parents at home, parent's education status, and the academic achievement of students. Table 2 shows the self-esteem levels and mean difference of the normal weight BMI and overweight BMI groups. We found a significant difference in means in the self-esteem levels of overweight students (*M* = 21.35, SD = 3.05) and normal weight students (*M* = 20.76, SD = 3.489); *t*(537) = −2.057, *p* = 0.040. Table 2 shows the self-esteem levels and mean difference of students whose parents are educated and uneducated. We found a significant difference in means of students' self-esteem levels whose parents are uneducated (*M* = 21.42, SD = 3.095) and educated (*M* = 20.67, SD = 3.405); *t*(537) = −2.622, *p* = 0.009. Table 2 shows the difference in means in the self-esteem levels of students who have a strict father or mother. We found a significant difference in means of the self-esteem levels of students whose father was strict (*M* = 20.99, SD = 3.169) and mother was strict (*M* = 21.41, SD = 3.33); *t*(537) = −1.45, *p* = 0.007. Table 2 shows the mean difference in the self-esteem levels of students with their academic performance. A significant difference in means was found in student's self-esteem levels who were average/below the average (*M* = 21.47, SD = 3.088) and above the average (*M* = 20.62, SD = 3.387); *t*(537) = −2.997, *p* = 0.003.

**Table 2:** Comparison of different variables on self-esteem levels among adolescents.

	Number	Mean	SD	df	*t*-value	*p* (sig-value)
**BMI**						
Normal weight	198	20.76	3.489	537	−2.057	0.04
Overweight	341	21.35	3.05			
**Parents’ education**						
Educated	201	20.67	3.405	537	−2.622	0.009
Uneducated	338	21.42	3.095			
**Parents’ strictness**						
Father	344	20.99	3.169	537	−1.45	0.007
Mother	195	21.41	3.33			
**Academic progress**						
Above the average	210	20.62	3.387	537	−2.997	0.003
Below the average	329	21.47	3.088			
**Gender**						
Male	232	21	3.289	537	−1.162	0.246
Female	307	21.32	3.15			
**Tuition**						
No	327	21.32	2.922	537	1.643	0.101
Yes	212	20.85	3.647			

We also performed an independent sample *t*-test for gender (male/female) and students who had tuition, as shown in Table 2, respectively. Our data showed no significant mean difference between genders (male and female) and students who did or did not have tuition.

## Discussion

This school-based study was designed to assess self-esteem among adolescents using RSES by measuring positive and negative feelings about the self.

Many factors influence the self-esteem of adolescents. Our present study found that weight, parents' education, the strictness of parents at home, and academic performance are significantly associated with self-esteem among adolescents in slum areas of Karachi, Pakistan.

Our study found that weight substantially impacted adolescents' self-esteem; hence, we accepted the H2 hypothesis. A study conducted by Sahin et al. in 2013 on the weight of Turkish children and adolescents also revealed that their weight condition significantly affects their overall self-perception (26). Similarly, a study by Gow ML et al. in California proved that self-esteem is lower in children and adolescents if they are overweight compared to peers with a healthy weight (27). According to Hamidreza Zakeri, a researcher from Iran, overweight children experience bullying at school and are more likely to develop poor self-esteem and depression (28).

Yılmazel and Günay (2012) and Gelbal et al. (2010) reported a positive association between parents' education status and the self-esteem of children and adolescents, influencing us to accept the H3 hypothesis. Likewise, our present research concluded that adolescent self-esteem increases with the increase in their parent's education level (29, 30). Our findings complement and expand upon those from earlier research. Conversely, Keskin (2010) concluded from a study that there were no appreciable variations in children's self-esteem scores based on the parent's education level (31). Moreover, in our study, most of the parents in the slum areas of Karachi were not educated enough. School-going children receive no study help from their parents which may indirectly impact their self-esteem. School-going adolescents also feel hesitant to take their uneducated parents to school meetings.

In addition, our study suggests that parenting practices also influence adolescent self-esteem. We concluded that adolescents whose fathers are stricter than their mothers experience mild to moderate levels of self-esteem. This may be because of Pakistani culture, which is a more male-dominant society, so the father in most families is the sole earner and stricter. Therefore, school-going youth behavior reveals their home environment. Girls in slum areas mostly stay at home; therefore, their self-esteem is affected more than boys. Khodarahimi S et al. (2011) also found in their study that daughters had low self-esteem if their fathers were strict (32). In terms of the connections between parenting styles and adolescent self-esteem, the dimensions of effect, communication, and sense of humor were found by Reina et al. to favorably impact self-esteem and life satisfaction in a representative sample of Spanish adolescents aged 12–17. In contrast, psychological control had the opposite effect (33). Hence we need to reject the H4 hypothesis.

Based on our study, academic performance is another predictor that contributes to adolescents' self-esteem development. According to Georgiou SN (2010), students encounter a wide range of self-referenced, task-related, and social emotions in academic contexts (34). Alam from California (2013) also pointed out that students who reported having more significant levels of self-esteem performed better academically when compared to students who reported having lower levels of self-esteem (35). Hence, we accept the H6 hypothesis.

In both developed and emerging countries, private tuition has constantly enhanced the traditional conventional education system (36). Some previous studies have also shown that tuition-going students could develop self-esteem and a sense of achievement because it helps them to keep up with their fellows and to expand their learning further (37). Our study did not support the idea of a self-esteem association with tuition-going and non-tuition-going adolescents. This may be due to less frequent practice of sending adolescents to tuition in slum areas because of low incomes among families. Surprisingly, this study found no gender differences in the levels of self-esteem. However, previous studies have shown that gender influences self-esteem among adolescents. In previous studies, girls constantly scored significantly lower on self-esteem measures than boys, highlighting the more adverse effects of low self-esteem in adolescent girls (38). Hence, we rejected H1 and H5 hypotheses as there was no impact of tuition and gender on the self-esteem of the school-going adolescents among our target population.

## Conclusion

Low self-esteem needs further investigation as a possible target for identifying adolescents at risk of internalizing psychopathologies. The RSES is a simple instrument and its midpoint may be used as a cutoff for low self-esteem, increasing its value in contexts such as schools. Teachers, parents, therapists, and others should concentrate on increasing self-esteem, assuming that high self-esteem will result in various benefits and advantages.

## Strengths of the study

This is the first study on adolescents in slum areas of Karachi. In the past, no studies were reported on children, especially in slums. A high level of self-esteem is fundamental for every child to progress in his/her future. This study will help to evaluate any mental health disorder in the early stages. The target population was highly vulnerable and, due to some limitations, these populations should have previously been addressed for such productive studies. This study can be carried out in other cities of Pakistan and among youth so we can quickly figure out the problem areas and work on their mental well-being.

## Limitations of the study

The data were collected within one month and could have been more significant and productive if we considered the data duration of at least three to six months. Moreover, in slum areas, the parents are not literate, so they are not aware of such kinds of screening, studies and knowledge; hence little reluctant to respond to some other questions and are least bothered about the outcome of this research. The schools in the slums area are community-based, where people are not interested and trust the people outside their community and not showing interest in sharing their information.

## Data Availability

The raw data used in this article are accessible from the corresponding author upon request.
